# Silent Retinal Neurodegeneration in Multiple Sclerosis: Structural Evidence from Clinically Unaffected Eyes Using Swept-Source OCT and OCT Angiography—A Cross-Sectional, Observational Study

**DOI:** 10.3390/biomedicines14071410

**Published:** 2026-06-23

**Authors:** Katarina Katanić Pasovski, Ivana Todorović, Viktor Pasovski, Dragana Ristić, Zorana Pavlović, Miloš Danilović, Nemanja Rančić, Tatjana Bošković Matić, Ranko Raičević, Evica Dinčić

**Affiliations:** 1 Clinic for Ophthalmology, Military Medical Academy, 11040 Belgrade, Serbia; dadana25@yahoo.com (D.R.); zoranapavlovic83@gmail.com (Z.P.); 2 Faculty of Medicine of the Military Medical Academy, University of Defense, 11040 Belgrade, Serbia; viktorpasovski@yahoo.com (V.P.); nece84@hotmail.com (N.R.); ranko.raicevic1@gmail.com (R.R.); evica.vma@gmail.com (E.D.); 3 Eye Clinic, Zvezdara University Medical Center, 11000 Belgrade, Serbia; ivana_j2003@yahoo.com; 4 Clinic for Neurology, Military Medical Academy, 11040 Belgrade, Serbia; drmilosdanilovic@gmail.com; 5 Center for Clinical Pharmacology, Military Medical Academy, 11040 Belgrade, Serbia; 6 Department of Neurology, Faculty of Medical Sciences, University of Kragujevac, 34000 Kragujevac, Serbia; stmatic769@gmail.com

**Keywords:** multiple sclerosis, optic neuritis, retinal neurodegeneration, swept-source optical coherence tomography, OCT angiography, ganglion cell–inner plexiform layer, superficial vascular plexus, visual pathway

## Abstract

**Background/Objectives:** Retinal optical coherence tomography (OCT) has emerged as a sensitive biomarker of neurodegeneration in multiple sclerosis (MS), yet eyes without overt optic neuritis (ON) are routinely pooled as “clinically unaffected” despite their heterogeneous histories. We evaluated whether never-ON eyes and fellow eyes after unilateral ON differ structurally and microvascularly using swept-source OCT (SS-OCT) and OCT angiography (OCTA). **Methods:** In this cross-sectional, single-center study, 126 clinically unaffected MS eyes—96 never-ON eyes and 30 fellow eyes after unilateral ON—were compared with 118 healthy control eyes. SS-OCT quantified ganglion cell–inner plexiform layer (GCIPL), peripapillary retinal nerve fiber layer (pRNFL), and macular RNFL (mRNFL) thickness, while OCTA measured superficial vascular plexus (SVP) vessel density. Between-group differences were assessed using generalized estimating equations with participant-level clustering, empirical (sandwich) standard errors, adjustment for age and sex, and false discovery rate correction. **Results:** Despite preserved visual acuity, both never-ON and fellow eyes showed structural retinal thinning relative to controls. GCIPL thickness followed a stepwise gradient—66.14 ± 4.31, 62.08 ± 7.03, and 58.03 ± 7.71 µm in controls, never-ON eyes, and fellow eyes, respectively (FDR-adjusted q = 0.020 for fellow vs. never-ON eyes)—and pRNFL and mRNFL showed a similar overall pattern. After false discovery rate correction, OCTA parameters did not differ significantly between groups. **Conclusions:** Clinically unaffected eyes in MS are not structurally normal, and fellow eyes after unilateral ON carry a greater burden of silent retinal damage than never-ON eyes. These two phenotypes should be analyzed separately in MS imaging research. Structural OCT measures, particularly GCIPL thickness, appear more sensitive than microvascular indices for detecting subclinical retinal involvement.

## 1. Introduction

Multiple sclerosis (MS) is an immune-mediated inflammatory and neurodegenerative disease of the central nervous system, characterized by focal demyelinating lesions as well as diffuse neuroaxonal injury that may evolve independently of overt inflammatory activity [1,2]. Increasing evidence suggests that progression of disability can occur in the absence of clinical relapses, a phenomenon often described as progression independent of relapse activity (PIRA), highlighting the need for sensitive biomarkers capable of capturing subtle and early neurodegenerative changes [3,4,5].

Optic neuritis (ON) is one of the most common clinical manifestations of visual-pathway involvement in MS and is frequently associated with persistent structural and functional deficits [6,7]. However, retinal neuroaxonal loss has also been demonstrated in eyes without a history of ON, suggesting that retinal involvement may occur silently, beyond clinically evident optic nerve inflammation [8,9,10]. In both clinical practice and research settings, eyes without prior ON are often grouped together as “clinically unaffected,” despite potentially heterogeneous underlying pathological mechanisms.

In particular, eyes of patients who have never experienced ON and fellow eyes of patients with unilateral ON are frequently analyzed as a single category [11,12]. This approach may overlook biologically meaningful differences, as a history of ON has been associated with a more aggressive disease course and a greater burden of neurodegeneration [13,14]. Whether fellow eyes can truly be considered equivalent to eyes from patients without any prior optic nerve involvement remains an open and clinically relevant question.

The retina, as an unmyelinated extension of the central nervous system, offers a unique opportunity to study neurodegenerative processes in vivo [15]. Optical coherence tomography (OCT) enables high-resolution quantification of retinal neuroaxonal layers and has emerged as a well-established and reproducible biomarker of neurodegeneration in MS [16,17,18]. In particular, thinning of the ganglion cell–inner plexiform layer (GCIPL) has been shown to correlate with global neurodegeneration and disability progression [19,20].

More recently, OCT angiography (OCTA) has allowed noninvasive assessment of the retinal microvasculature, providing complementary information on vascular and metabolic alterations within the retina [21,22,23]. Although microvascular changes have been reported in MS, their relationship to structural retinal damage and their role in subclinical disease stages remain incompletely understood.

The primary aim of this study was to determine whether clinically unaffected eyes in MS represent a homogeneous category by comparing never-ON eyes and fellow eyes after unilateral ON using swept-source OCT and OCT angiography.

## 2. Materials and Methods

### 2.1. Study Design and Participants

This observational, cross-sectional study was conducted at a tertiary referral center and included consecutive patients with multiple sclerosis examined between January 2022 and January 2023. MS was diagnosed according to the 2017 revised McDonald criteria [24].

Only clinically unaffected eyes were analyzed. A clinically unaffected eye was defined as an eye without a history of ON and without clinical signs of acute or previous optic nerve involvement at the time of examination. Based on ON history, clinically unaffected eyes were assigned to one of two predefined groups: (i) fellow eyes—the contralateral eyes of MS patients with documented unilateral ON; and (ii) never-ON eyes—eyes of MS patients with no history of ON in either eye.

A group of healthy adults without neurological or ophthalmological disease was recruited as controls. Because age and sex distributions differed across groups, both age and sex were included as covariates in retinal-parameter models.

### 2.2. Inclusion and Exclusion Criteria

Inclusion criteria for MS patients were: confirmed diagnosis of MS; absence of clinical ON in the analyzed eye; best-corrected visual acuity (BCVA) ≥ 0.8 (decimal notation); and clinical stability at examination. Exclusion criteria were: a history of ON in the analyzed eye; other optic neuropathies; retinal or ocular disease affecting the optic nerve or macula; high refractive error (>±5 dioptres); media opacities interfering with image quality; and MS relapse or corticosteroid treatment within three months prior to examination. Healthy controls met standard eligibility criteria—absence of neurological or ophthalmological disease and no ocular condition affecting retinal structure or image quality.

### 2.3. Ophthalmological and Functional Assessment

All participants underwent a standardized ophthalmological examination including BCVA assessment with Snellen charts. Visual-pathway function was further evaluated using monocular pattern-reversal visual evoked potential (VEP), recorded with a standardized laboratory protocol. P100 latency and amplitude were analyzed for each eye. The laboratory upper limit of normal for P100 latency was 115 ms.

### 2.4. SS-OCT Imaging

Structural retinal imaging was performed using a swept-source optical coherence tomography device (DRI OCT Triton Plus; Topcon Corporation, Tokyo, Japan) with IMAGEnet 6 for Triton software, Version 1.04E (1.36.22755). The device operates at a 1050 nm center wavelength. Macular scans were acquired using the 3D Macula 7.0 × 7.0 mm protocol with a 512 × 256 sampling density and were analyzed within a 6 × 6 mm area. Macular retinal nerve fiber layer (mRNFL) and ganglion cell–inner plexiform layer thickness (GCIPL; Topcon GCL+) values were recorded for the total, superior, and inferior regions. Peripapillary retinal nerve fiber layer (pRNFL) thickness was derived from the 3D Wide 12.0 × 9.0 mm protocol with a 512 × 256 sampling density and measured along a 3.4 mm diameter peripapillary circle; global and quadrant values were recorded. Structural scans were processed in Fine analysis mode (2.0.7). Automated segmentation was reviewed manually to identify segmentation errors. During acquisition, participants fixated on the device’s built-in internal fixation target, displayed as a green cross. The device incorporates the SMARTTrack™ eye-tracking system; however, automated eye tracking was not systematically used and was disabled during most acquisitions. Scan position and centration were therefore adjusted manually by the operator using the device joystick and verified during acquisition.

### 2.5. OCT Angiography

OCT angiography (OCTA) was performed using the same device and the Angio Macula 3.0 × 3.0 mm (X4) protocol, centered on the fovea, with a 320 × 320 sampling density. Analysis focused on the automated superficial angiography slab, extending from ILM + 2.6 µm to IPL/INL + 15.6 µm. Vessel density was recorded for the superior, inferior, temporal, nasal, and central foveal (C-FOV) fields. Total vessel density was calculated as the arithmetic mean of these five values. The C-FOV value represents central foveal vessel density and not foveal avascular zone area. Deep vascular plexus parameters were not analyzed because reliable, validated, and reproducible quantitative deep-plexus vessel-density output was not available in the installed IMAGEnet 6 for Triton software configuration used for this dataset; therefore, the microvascular analysis was restricted to the automated superficial vascular plexus parameters.

### 2.6. Image Quality Control

All OCT and OCTA images were assessed for image quality, segmentation errors, motion artifacts, and scan centration. Scans judged to be of insufficient quality were excluded. Quality control was performed by experienced examiners during review of the imaging data.

### 2.7. Statistical Analysis

Statistical analyses were performed using Python 3.13.5 with SciPy 1.17.0 and statsmodels 0.14.6. Normality of continuous-variable distributions was assessed using the Shapiro–Wilk test. Continuous variables are presented as mean ± standard deviation or median [interquartile range], as appropriate. Categorical variables were compared using the chi-square test. Disease duration, EDSS, and MSSS were compared between the two MS subgroups using two-sided Mann–Whitney U tests. For retinal structural and OCTA parameters, between-group differences were assessed using generalized estimating equations (GEEs) with a Gaussian distribution, identity link, exchangeable working correlation structure, and empirical (sandwich) standard errors. Participant identifier was specified as the clustering variable to account for inter-eye correlation in individuals contributing both eyes. Age and sex were included as covariates. Pairwise contrasts were evaluated using Benjamini–Hochberg false discovery rate (FDR) correction across all pairwise contrasts within each prespecified parameter family. Two-sided FDR-adjusted q-values < 0.05 were considered statistically significant. No missing values were imputed; outcome-specific available-case analyses were used. Sensitivity analyses were additionally performed using linear mixed-effects models with a participant-level random intercept, analyses restricted to the common age range, and exact 1:1 matching for sex with an age caliper of up to 3 years to examine the stability of the findings across alternative analytical approaches.

This exploratory single-center study analyzed a consecutive cohort examined during the predefined study period; no a priori sample-size calculation determined recruitment. To aid interpretation of non-significant comparisons, an exploratory precision analysis was performed for selected fellow-versus-never-ON contrasts. The minimum detectable effect (MDE) for 80% power at a two-sided alpha level of 0.05 was calculated as (z0.975 + z0.80) × SE of the adjusted GEE contrast. MDE values are reported in Supplementary Table S4 and should not be interpreted as evidence of equivalence between groups.

### 2.8. Ethical Considerations

The study was conducted in accordance with the Declaration of Helsinki. The study protocol was approved by the Ethics Committee of the Military Medical Academy, Belgrade (Approval No. 6/2020, 4 August 2020). All participants provided written informed consent prior to inclusion.

## 3. Results

### 3.1. Study Population and Clinical Characteristics

The primary analysis included 126 clinically unaffected MS eyes (96 never-ON eyes and 30 fellow eyes after unilateral ON) and 118 healthy control eyes. Never-ON eyes were contributed by 48 MS patients without a history of ON, while one clinically unaffected fellow eye was analyzed for each of 30 patients with documented unilateral ON. Age and sex distributions differed across groups; therefore, both variables were included as covariates in retinal-parameter models. Disease duration and disability measures (Expanded Disability Status Scale—EDSS, Multiple Sclerosis Severity Score—MSSS) were similar between the two MS subgroups (Table 1). Best-corrected visual acuity was preserved in all clinically unaffected eyes included in the analysis.

### 3.2. Ganglion Cell–Inner Plexiform Layer Thickness

**Figure 1:**
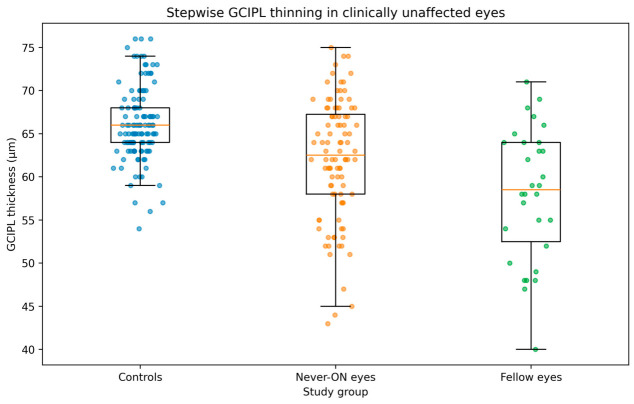
Stepwise GCIPL thinning in clinically unaffected eyes. The box plot shows the median and interquartile range; individual eye-level observations are overlaid. The central line indicates the median, boxes indicate the interquartile range, and whiskers extend to 1.5 × the interquartile range. Between-group q-values corresponding to age- and sex-adjusted GEE contrasts with participant-level clustering are reported in Table 2. GCIPL, ganglion cell–inner plexiform layer; ON, optic neuritis.

Age- and sex-adjusted GEE models confirmed a stepwise pattern of GCIPL thinning (Table 2, Figure 1). Mean GCIPL thickness was lower in never-ON eyes than in controls and was further reduced in fellow eyes after unilateral ON. This pattern remained statistically significant after FDR correction.

### 3.3. Peripapillary Retinal Nerve Fiber Layer Thickness

Global pRNFL thickness was reduced in clinically unaffected MS eyes compared with controls (Table 2). Fellow eyes showed the greatest global pRNFL reduction. Sectoral contrasts were less uniform after FDR correction: temporal thinning remained significant in both clinically unaffected MS-eye groups relative to controls, while several additional sectoral differences were observed mainly in fellow eyes.

### 3.4. Macular Retinal Nerve Fiber Layer Thickness

Macular RNFL measurements followed the same overall gradient as peripapillary measurements (Table 2). Both never-ON and fellow eyes showed lower global mRNFL values than controls. Fellow eyes showed numerically lower inferior and mean mRNFL values than never-ON eyes; however, neither fellow-versus-never-ON contrast reached statistical significance after FDR correction (q = 0.056 and q = 0.062, respectively). Central macular thickness did not differ between never-ON and fellow eyes (Supplementary Table S2), supporting a layer-specific rather than global macular-thickness pattern.

### 3.5. OCT Angiography Findings

**Figure 2:**
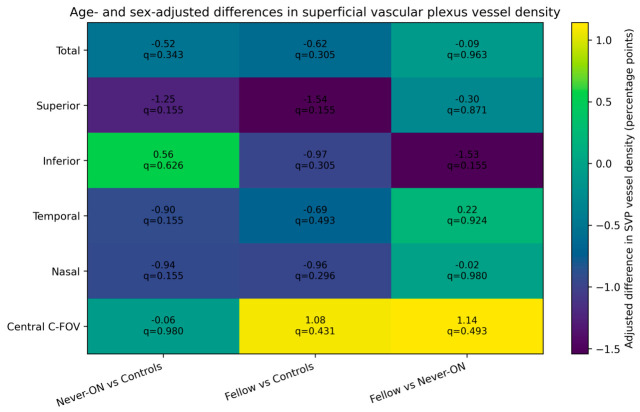
Age- and sex-adjusted differences in superficial vascular plexus vessel density across study groups. Values represent adjusted between-group differences in percentage points derived from GEE models with participant-level clustering and adjustment for age and sex. q-values are Benjamini–Hochberg FDR-adjusted within the OCTA family. C-FOV, central foveal vessel density; OCTA, optical coherence tomography angiography; SVP, superficial vascular plexus.

OCTA analysis of the superficial vascular plexus is summarized in Table 3 and Figure 2. Total vessel density was standardized as the arithmetic mean of the superior, inferior, temporal, nasal, and central foveal C-FOV fields. After adjustment for age and sex and FDR correction within the OCTA family, no OCTA parameter differed significantly between groups. OCTA findings should therefore be interpreted as exploratory and less consistent than the structural OCT findings. Age- and sex-adjusted estimated marginal means and contrasts for structural OCT and OCTA parameters are provided in Supplementary Table S3. Sensitivity analyses using mixed-effects models, restriction to the common age range, and sex- and age-matched subsamples supported the direction and overall interpretation of the principal structural OCT findings, whereas the small OCTA contrasts remained less consistent.

### 3.6. Functional Measures

Despite the structural retinal changes described above, BCVA remained preserved and did not differ significantly between fellow and never-ON eyes (Supplementary Table S2). In available-case analyses (76 never-ON eyes and 25 fellow eyes), P100 latency was 122.47 ± 18.40 ms in never-ON eyes and 128.84 ± 12.44 ms in fellow eyes (q = 0.290). VEP amplitude was 7.13 ± 3.91 µV in never-ON eyes and 7.07 ± 3.48 µV in fellow eyes (q = 0.863). Mean P100 latency was prolonged in both clinically unaffected MS-eye groups relative to the laboratory upper limit of normal (115 ms), while the between-group difference was not statistically significant. These findings indicate subclinical visual-pathway dysfunction in both MS-eye phenotypes, although VEP did not distinguish fellow from never-ON eyes as clearly as structural OCT.

## 4. Discussion

Using swept-source OCT and OCT angiography, we found that clinically unaffected eyes in MS are not structurally spared. When never-ON eyes were analyzed separately from fellow eyes after unilateral ON, distinct structural differences emerged despite preserved visual acuity: fellow eyes showed more pronounced retinal neuroaxonal loss than never-ON eyes, most clearly evidenced by GCIPL thinning. These findings remained evident after accounting for inter-eye correlation, age, sex, and multiple comparisons. Together, the results indicate that retinal involvement in MS extends beyond clinically affected eyes and may reflect a diffuse, silent neurodegenerative component of the disease [3,4,8,9,10].

The detection of retinal thinning in never-ON eyes supports the view that subclinical retinal neuroaxonal loss can occur in the absence of clinically apparent inflammatory events. Structural OCT showed measurable reductions relative to controls, suggesting that retinal involvement can develop without an overt clinical episode. Previous studies have linked such OCT changes to broader neurodegeneration in MS, including brain atrophy and disability outcomes, and have highlighted GCIPL thickness as a sensitive marker of early, “silent” tissue loss. In our cohort, GCIPL showed the most consistent differences between the clinically unaffected eye phenotypes, supporting its practical value as a biomarker of subclinical retinal involvement [10,17,18,19,20,25].

Arguably the most relevant finding is that fellow eyes after unilateral ON showed a greater degree of retinal neuroaxonal loss than never-ON eyes, again most clearly as more pronounced GCIPL thinning. Although both groups were clinically unaffected at examination, fellow eyes had lower GCIPL values despite comparable EDSS and MSSS, suggesting that prior ON is associated with greater subclinical contralateral retinal damage that is not necessarily captured by global clinical disability measures. Previous studies have reported retinal abnormalities in fellow eyes after unilateral ON and have suggested bilateral or trans-synaptic effects [12,26,27]. Our study differs by directly separating two clinically unaffected phenotypes—never-ON eyes and fellow eyes after unilateral ON—within a predefined analytical framework, by combining structural SS-OCT with OCTA, and by applying participant-level models that account for inter-eye correlation, age, sex, and multiple comparisons. The resulting structural gradient supports the view that fellow eyes should not be pooled indiscriminately with never-ON eyes in retinal-imaging studies.

In our cohort, structural OCT appeared more informative than OCTA for detecting subclinical retinal involvement in clinically unaffected eyes. GCIPL and RNFL thinning remained consistent across several comparisons after FDR correction, whereas no OCTA parameter retained statistical significance. These findings suggest that microvascular alterations may be more subtle, heterogeneous, or technically sensitive than structural neuroaxonal loss in clinically unaffected eyes. Overall, the results support GCIPL thickness as a particularly sensitive structural marker of diffuse retinal involvement in MS [10,18,21,22,28,29,30].

A further key observation is the partial dissociation between structural retinal injury and routinely measured visual function. BCVA remained preserved across clinically unaffected eye groups. However, mean P100 latency was prolonged in both MS-eye phenotypes relative to the laboratory upper limit of normal (115 ms), while the difference between fellow and never-ON eyes was not statistically significant. Thus, VEP suggests subclinical visual-pathway dysfunction in both groups, whereas GCIPL thickness provides a clearer structural separation between the two clinically unaffected phenotypes. This pattern is compatible with early or subclinical visual-system involvement in MS [10,31,32].

These results also have implications for the design and interpretation of retinal-imaging studies in MS. Treating all clinically unaffected eyes as a single group may obscure differences related to prior ON and to overall disease burden. In our cohort, fellow eyes after unilateral ON exhibited more silent retinal damage even when visual acuity was preserved; for research, this argues for analyzing never-ON and fellow eyes separately when retinal imaging is used as an outcome or biomarker. In clinical practice, structural retinal assessment—particularly GCIPL thickness—may add useful information beyond standard clinical and functional testing [18,33].

Several limitations should be acknowledged. First, this was an observational, cross-sectional, single-center study conducted in a tertiary-referral setting, which limits causal inference, may reduce generalizability, and introduces the possibility of selection bias toward patients with more complex or closely monitored disease. Second, the cross-sectional design prevents assessment of temporal changes in retinal structural or microvascular parameters and their relationship to subsequent clinical progression. Third, retinal-imaging findings could not be correlated with MRI measures, including brain atrophy or lesion burden, limiting our ability to determine how retinal neurodegeneration relates to broader central nervous system damage. Fourth, age and sex distributions differed across groups; although both variables were included as covariates, residual confounding cannot be fully excluded. Fifth, OCTA measurements can be influenced by technical and methodological factors, including image quality, segmentation accuracy, motion artifacts, and scan protocol, which may reduce sensitivity to subtle microvascular differences. Finally, the OCTA analysis was limited to superficial macular microvascular parameters because reliable quantitative deep vascular plexus output was not available in the installed software workflow used for this dataset. Therefore, deeper plexus involvement cannot be excluded, and the microvascular interpretation should be considered limited to superficial vascular plexus parameters. Future multicenter longitudinal studies integrating retinal imaging with MRI, clinical, radiological, functional, and multilayer OCTA outcomes are needed to better define the prognostic value of silent retinal neurodegeneration in MS [17,18,25,28,32,34]. The study was not prospectively powered for all null contrasts, particularly the smaller fellow-eye subgroup and OCTA comparisons; accordingly, exploratory MDE values are provided in Supplementary Table S4, and non-significant findings should not be interpreted as proof of equivalence.

## 5. Conclusions

Clinically unaffected eyes in MS are not truly spared. We showed that silent retinal neurodegeneration is already present in eyes without prior optic neuritis, even when visual acuity is preserved. Importantly, fellow eyes after unilateral ON had more pronounced structural retinal damage than never-ON eyes, suggesting that these two “clinically unaffected” phenotypes are not equivalent and likely reflect different underlying disease burden.

Structural OCT measures, especially GCIPL thickness, were more sensitive than OCTA metrics in detecting subclinical involvement, supporting SS-OCT as a practical marker of diffuse neurodegeneration. OCTA findings were less consistent after correction for multiple comparisons and should be interpreted as exploratory. The combination of measurable structural loss, preserved visual acuity, and prolonged mean P100 latency suggests that conventional clinical measures may underestimate early tissue damage.

Overall, our results highlight the importance of careful phenotyping of clinically unaffected eyes in both research and clinical practice, and they support retinal imaging as a sensitive tool for detecting subclinical neurodegenerative changes in MS.

## Figures and Tables

**Table 1:** Demographic and clinical characteristics of the study groups.

Characteristic	Never-ON Eyes (MS Without ON)	Fellow Eyes (After Unilateral ON)	Control Subjects	*p*-Value
Participants, *n* (eyes)	48 (96)	30 (30) ^a^	59 (118)	–
Sex, male/female	28 (58.3%)/20 (41.7%)	11 (36.7%)/19 (63.3%)	20 (33.9%)/39 (66.1%)	0.029
Age, years (mean ± SD)	44.6 ± 7.4	40.1 ± 11.1	37.2 ± 9.7	<0.001
Disease duration since first symptoms, years (median [IQR])	11.5 [6.8–15.0]	11.5 [5.5–13.0]	–	0.551
Disease duration since diagnosis, years (median [IQR])	10.0 [5.0–12.2]	9.0 [4.2–12.0]	–	0.688
EDSS (median [IQR])	2.0 [1.0–4.5]	1.5 [1.0–3.1]	–	0.391
MSSS (median [IQR])	3.34 [1.03–4.80]	2.44 [1.34–4.34]	–	0.617

^a^ One clinically unaffected fellow eye contributed per patient with documented unilateral ON. Sex distribution: chi-square test. Age: one-way ANOVA; Kruskal–Wallis sensitivity *p* = 0.001. Disease duration, EDSS, and MSSS: two-sided Mann–Whitney U tests comparing the two MS subgroups. Available-case analyses were used for EDSS (47 never-ON and 28 fellow-eye participants) and MSSS (47 never-ON and 27 fellow-eye participants). Age and sex were included as covariates in retinal-parameter models. EDSS, Expanded Disability Status Scale; IQR, interquartile range; MS, multiple sclerosis; MSSS, Multiple Sclerosis Severity Score; ON, optic neuritis; SD, standard deviation.

**Table 2:** Structural OCT parameters in clinically unaffected eyes.

Parameter	Never-ON Eyes	Fellow Eyes	Controls	q (N vs. C)	q (F vs. C)	q (F vs. N)
**GCIPL mean (µm)**	**62.08 ± 7.03**	**58.03 ± 7.71**	**66.14 ± 4.31**	**0.002**	**<0.001**	**0.020**
GCIPL superior (µm)	62.44 ± 7.27	58.83 ± 7.80	66.53 ± 4.75	0.002	<0.001	0.031
GCIPL inferior (µm)	61.58 ± 6.96	57.97 ± 6.85	65.87 ± 4.07	<0.001	<0.001	0.027
**pRNFL total (µm)**	**101.14 ± 14.76**	**90.37 ± 19.88**	**107.77 ± 8.58**	**0.022**	**<0.001**	**0.015**
pRNFL superior (µm)	126.71 ± 19.55	110.40 ± 25.67	132.83 ± 11.39	0.073	<0.001	0.004
pRNFL inferior (µm)	127.54 ± 21.84	116.00 ± 28.39	138.26 ± 15.26	0.015	<0.001	0.054
pRNFL nasal (µm)	78.15 ± 14.20	71.23 ± 17.77	84.03 ± 13.25	0.052	0.001	0.058
pRNFL temporal (µm)	68.38 ± 17.99	65.30 ± 23.62	76.69 ± 9.84	0.004	0.015	0.626
**mRNFL mean (µm)**	**37.22 ± 5.51**	**33.93 ± 9.02**	**40.92 ± 5.49**	**<0.001**	**<0.001**	**0.062**
mRNFL superior (µm)	35.28 ± 5.03	32.47 ± 9.43	39.63 ± 5.96	<0.001	<0.001	0.105
mRNFL inferior (µm)	39.03 ± 6.40	35.07 ± 9.99	42.12 ± 5.82	0.015	<0.001	0.056

Values are unadjusted mean ± SD for descriptive purposes. q-values are Benjamini–Hochberg FDR-adjusted *p*-values across all pairwise contrasts within each prespecified structural parameter family. Pairwise contrasts were obtained from GEE models with participant as the clustering variable, exchangeable working correlation, empirical (sandwich) standard errors, and adjustment for age and sex. Age- and sex-adjusted estimated marginal means, adjusted contrasts, and 95% confidence intervals are provided in Supplementary Table S3. Outcome-specific available-case analyses were used; one fellow-eye inferior mRNFL value was missing. Bold rows indicate mean/global parameters. C, controls; F, fellow eyes; GCIPL, ganglion cell–inner plexiform layer; mRNFL, macular retinal nerve fiber layer; N, never-ON eyes; ON, optic neuritis; pRNFL, peripapillary retinal nerve fiber layer.

**Table 3:** OCT angiography (superficial vascular plexus) measures in clinically unaffected eyes.

Parameter	Never-ON Eyes	Fellow Eyes	Controls	q (N vs. C)	q (F vs. C)	q (F vs. N)
**Total vessel density (%)**	**42.21 ± 2.36**	**42.06 ± 2.14**	**42.70 ± 1.37**	**0.343**	**0.305**	**0.963**
Superior (%)	48.80 ± 3.48	48.65 ± 3.16	50.28 ± 2.52	0.155	0.155	0.871
Inferior (%)	49.42 ± 4.85	48.27 ± 2.74	49.39 ± 3.73	0.626	0.305	0.155
Temporal (%)	46.62 ± 2.90	46.69 ± 3.72	47.37 ± 2.12	0.155	0.493	0.924
Nasal (%)	45.85 ± 3.35	45.67 ± 2.98	46.58 ± 2.15	0.155	0.296	0.980
Central foveal vessel density, C-FOV (%)	20.37 ± 5.24	21.02 ± 4.44	19.87 ± 4.56	0.980	0.431	0.493

Values are unadjusted mean ± SD for descriptive purposes. q-values are Benjamini–Hochberg FDR-adjusted *p*-values across all pairwise contrasts within the OCTA family. Pairwise contrasts were obtained from GEE models with participant as the clustering variable, exchangeable working correlation, empirical (sandwich) standard errors, and adjustment for age and sex. Age- and sex-adjusted estimated marginal means, adjusted contrasts, and 95% confidence intervals are provided in Supplementary Table S3. Total vessel density was calculated as the arithmetic mean of superior, inferior, temporal, nasal, and central C-FOV vessel-density values. C-FOV denotes central foveal vessel density and should not be labeled as FAZ area. The bold row indicates the total vessel-density summary measure. C, controls; C-FOV, central foveal vessel density; F, fellow eyes; FAZ, foveal avascular zone; FDR, false discovery rate; GEE, generalized estimating equations; N, never-ON eyes; OCTA, optical coherence tomography angiography; ON, optic neuritis; SD, standard deviation.

## Data Availability

The data presented in this study are available on request from the corresponding author. The data are not publicly available due to privacy and ethical restrictions related to clinical patient information.

## Supplementary Materials

The following supporting information can be downloaded at https://www.mdpi.com/article/10.3390/biomedicines14071410/s1. Supplementary Table S1: Structural OCT and OCTA parameters in ON-affected eyes; Supplementary Table S2: Extended structural OCT, OCTA, and functional parameters—comparison of clinically unaffected eyes; Supplementary Table S3: Age- and sex-adjusted GEE results for structural OCT and OCTA parameters in clinically unaffected eyes; Supplementary Table S4: Exploratory minimum detectable effect analysis for selected fellow-versus-never-ON contrasts.
