# Supplementary material for: Silent Retinal Neurodegeneration in Multiple Sclerosis: Structural Evidence from Clinically Unaffected Eyes Using Swept-Source OCT and OCT Angiography—A Cross-Sectional, Observational Study
Source: Biomedicines. 2026 Jun 23;14(7):1410. doi: 10.3390/biomedicines14071410 (PMC13405330; doi:10.3390/biomedicines14071410)
Supplement: Supplementary file 1 [file biomedicines-14-01410-s001.zip › biomedicines-4366745-SI.pdf]

Supplementary Materials

Silent Retinal Neurodegeneration in Multiple Sclerosis: Structural Evidence from Clinically Unaffected Eyes Using Swept-Source OCT and OCT Angiography – A Cross-Sectional, Observational Study

Supplementary Table S1. Structural OCT and OCTA parameters in ON-affected eyes.

Parameter	ON-affected eyes (MS with unilateral ON) (n = 30 eyes)	Control subjects (n = 118 eyes)	q (ON-affected vs Controls)
GCIPL mean (µm)	53.87 ± 8.97	66.14 ± 4.31	**<0.001**
GCIPL superior (µm)	54.87 ± 9.13	66.53 ± 4.75	**<0.001**
GCIPL inferior (µm)	52.87 ± 8.92	65.87 ± 4.07	**<0.001**
pRNFL total (µm)	82.20 ± 20.39	107.77 ± 8.58	**<0.001**
pRNFL superior (µm)	103.80 ± 22.78	132.83 ± 11.39	**<0.001**
pRNFL inferior (µm)	105.93 ± 30.70	138.26 ± 15.26	**<0.001**
pRNFL nasal (µm)	68.20 ± 19.38	84.03 ± 13.25	**<0.001**
pRNFL temporal (µm)	56.43 ± 19.45	76.69 ± 9.84	**<0.001**
mRNFL mean (µm)	31.20 ± 7.61	40.92 ± 5.49	**<0.001**
mRNFL superior (µm)	29.53 ± 6.88	39.63 ± 5.96	**<0.001**
mRNFL inferior (µm)	33.13 ± 8.95	42.12 ± 5.82	**<0.001**
OCTA total vessel density (%)	40.58 ± 2.60	42.70 ± 1.37	**<0.001**
OCTA superior (%)	47.21 ± 4.37	50.28 ± 2.52	**<0.001**
OCTA inferior (%)	46.14 ± 3.98	49.39 ± 3.73	**<0.001**
OCTA temporal (%)	44.36 ± 3.26	47.37 ± 2.12	**<0.001**
OCTA nasal (%)	44.17 ± 3.64	46.58 ± 2.15	**<0.001**
OCTA central foveal vessel density, C-FOV (%)	21.01 ± 4.46	19.87 ± 4.56	0.279

Values are presented as mean ± SD. q-values are Benjamini–Hochberg false discovery rate (FDR)-adjusted p-values across all tested contrasts within each prespecified parameter family. Between-group contrasts were obtained from generalized estimating equation (GEE) models with participant identifier as the clustering variable, an exchangeable working correlation structure, empirical (sandwich) standard errors, and adjustment for age and sex. Total OCTA vessel density was calculated as the arithmetic mean of the superior, inferior, temporal, nasal, and central C-FOV vessel-density values. C-FOV denotes central foveal vessel density and not foveal avascular zone area. GCIPL, ganglion cell–inner plexiform layer; mRNFL, macular retinal nerve fiber layer; MS, multiple sclerosis; OCT, optical coherence tomography; OCTA, optical coherence tomography angiography; ON, optic neuritis; pRNFL, peripapillary retinal nerve fiber layer; SD, standard deviation.

Supplementary Table S2. Extended structural OCT, OCTA, and functional parameters – comparison of clinically unaffected eyes.

Parameter	Never-ON eyes (MS without ON) (n = 96 eyes)	Fellow eyes (after unilateral ON) (n = 30 eyes)	q (Fellow vs Never-ON)
pRNFL total (µm)	101.14 ± 14.76	90.37 ± 19.88	**0.015**
pRNFL superior (µm)	126.71 ± 19.55	110.40 ± 25.67	**0.004**
pRNFL inferior (µm)	127.54 ± 21.84	116.00 ± 28.39	0.054
pRNFL nasal (µm)	78.15 ± 14.20	71.23 ± 17.77	0.058
pRNFL temporal (µm)	68.38 ± 17.99	65.30 ± 23.62	0.626
CMT (µm)	236.60 ± 19.17	233.57 ± 17.64	0.981
mRNFL superior (µm)	35.28 ± 5.03	32.47 ± 9.43	0.105
mRNFL inferior (µm)	39.03 ± 6.40	35.07 ± 9.99	0.056
mRNFL mean (µm)	37.22 ± 5.51	33.93 ± 9.02	0.062
GCIPL superior (µm)	62.44 ± 7.27	58.83 ± 7.80	**0.031**
GCIPL inferior (µm)	61.58 ± 6.96	57.97 ± 6.85	**0.027**
GCIPL mean (µm)	62.08 ± 7.03	58.03 ± 7.71	**0.020**
OCTA superior (%)	48.80 ± 3.48	48.65 ± 3.16	0.871
OCTA inferior (%)	49.42 ± 4.85	48.27 ± 2.74	0.155
OCTA temporal (%)	46.62 ± 2.90	46.69 ± 3.72	0.924
OCTA nasal (%)	45.85 ± 3.35	45.67 ± 2.98	0.980
OCTA total vessel density (%)	42.21 ± 2.36	42.06 ± 2.14	0.963
OCTA central foveal vessel density, C-FOV (%)	20.37 ± 5.24	21.02 ± 4.44	0.493
Best-corrected visual acuity (decimal)	0.98 ± 0.08	0.99 ± 0.03	0.290
VEP latency (ms)	122.47 ± 18.40	128.84 ± 12.44	0.290
VEP amplitude (µV)	7.13 ± 3.91	7.07 ± 3.48	0.863

Values are presented as mean ± SD. q-values are Benjamini–Hochberg FDR-adjusted p-values across all tested contrasts within each prespecified parameter family. Structural and OCTA contrasts correspond to age- and sex-adjusted GEE models with participant-level clustering, an exchangeable working correlation structure, and empirical (sandwich) standard errors. Functional variables were analyzed analogously. CMT was treated as a standalone structural parameter. No missing values were imputed; outcome-specific available-case analysis was used. One inferior mRNFL value was missing in the fellow-eye group. For VEP parameters, available-case sample sizes were 76 never-ON eyes and 25 fellow eyes. Total OCTA vessel density was calculated as the arithmetic mean of the superior, inferior, temporal, nasal, and central C-FOV vessel-density values. BCVA, best-corrected visual acuity; C-FOV, central foveal vessel density; CMT, central macular thickness; GCIPL, ganglion cell–inner plexiform layer; mRNFL, macular retinal nerve fiber layer; MS, multiple sclerosis; OCT, optical coherence tomography; OCTA, optical coherence tomography angiography; ON, optic neuritis; pRNFL, peripapillary retinal nerve fiber layer; SD, standard deviation; VEP, visual evoked potential.

Supplementary Table S3. Age- and sex-adjusted GEE results for structural OCT and OCTA parameters in clinically unaffected eyes.

Family	Parameter	Adjusted mean Controls [95% CI]	Adjusted mean Never-ON [95% CI]	Adjusted mean Fellow [95% CI]	Adjusted difference N-C [95% CI]	q N-C	Adjusted difference F-C [95% CI]	q F-C	Adjusted difference F-N [95% CI]	q F-N
GCIPL	GCIPL mean (µm)	66.10 [64.92, 67.28]	62.10 [60.16, 64.04]	58.15 [55.57, 60.72]	-4.00 [-6.37, -1.63]	0.002	-7.95 [-10.82, -5.09]	<0.001	-3.95 [-7.15, -0.76]	0.020
GCIPL	GCIPL superior (µm)	66.46 [65.21, 67.71]	62.49 [60.51, 64.47]	58.93 [56.31, 61.54]	-3.97 [-6.39, -1.56]	0.002	-7.53 [-10.47, -4.59]	<0.001	-3.56 [-6.80, -0.32]	0.031
GCIPL	GCIPL inferior (µm)	65.89 [64.79, 66.99]	61.53 [59.61, 63.44]	58.09 [55.77, 60.42]	-4.36 [-6.66, -2.06]	<0.001	-7.80 [-10.41, -5.19]	<0.001	-3.44 [-6.42, -0.45]	0.027
pRNFL	pRNFL total (µm)	107.57 [104.97, 110.18]	101.33 [97.33, 105.32]	90.53 [83.75, 97.31]	-6.25 [-11.27, -1.23]	0.022	-17.04 [-24.33, -9.76]	<0.001	-10.79 [-18.64, -2.95]	0.015
pRNFL	pRNFL superior (µm)	132.68 [129.41, 135.94]	126.81 [121.77, 131.84]	110.69 [101.96, 119.42]	-5.87 [-12.19, 0.44]	0.073	-21.99 [-31.31, -12.67]	<0.001	-16.12 [-26.18, -6.05]	0.004
pRNFL	pRNFL inferior (µm)	137.97 [133.65, 142.30]	127.86 [122.06, 133.65]	116.14 [106.37, 125.90]	-10.12 [-17.72, -2.51]	0.015	-21.84 [-32.50, -11.17]	<0.001	-11.72 [-23.06, -0.37]	0.054
pRNFL	pRNFL nasal (µm)	83.83 [80.56, 87.09]	78.39 [74.62, 82.16]	71.28 [65.17, 77.40]	-5.44 [-10.59, -0.29]	0.052	-12.54 [-19.48, -5.60]	0.001	-7.10 [-14.22, 0.02]	0.058
pRNFL	pRNFL temporal (µm)	76.96 [74.48, 79.44]	67.97 [63.20, 72.75]	65.55 [57.23, 73.87]	-8.99 [-14.53, -3.44]	0.004	-11.41 [-19.93, -2.89]	0.015	-2.42 [-12.15, 7.30]	0.626
mRNFL	mRNFL mean (µm)	40.84 [39.58, 42.09]	37.31 [35.85, 38.77]	33.97 [30.87, 37.08]	-3.53 [-5.52, -1.53]	<0.001	-6.86 [-10.22, -3.51]	<0.001	-3.34 [-6.75, 0.07]	0.062
mRNFL	mRNFL superior (µm)	39.52 [38.16, 40.88]	35.40 [34.03, 36.77]	32.51 [29.27, 35.75]	-4.12 [-6.11, -2.12]	<0.001	-7.01 [-10.53, -3.49]	<0.001	-2.89 [-6.39, 0.60]	0.105
mRNFL	mRNFL inferior (µm)	42.02 [40.70, 43.33]	39.15 [37.50, 40.80]	35.08 [31.55, 38.62]	-2.86 [-5.05, -0.68]	0.015	-6.93 [-10.65, -3.22]	<0.001	-4.07 [-8.03, -0.11]	0.056
OCTA	SVP total vessel density (%)	42.71 [42.36, 43.07]	42.19 [41.59, 42.79]	42.09 [41.34, 42.85]	-0.52 [-1.28, 0.23]	0.343	-0.62 [-1.43, 0.19]	0.305	-0.09 [-1.09, 0.90]	0.963
OCTA	SVP superior vessel density (%)	50.18 [49.55, 50.81]	48.93 [48.09, 49.78]	48.64 [47.54, 49.73]	-1.25 [-2.38, -0.11]	0.155	-1.54 [-2.81, -0.27]	0.155	-0.30 [-1.70, 1.10]	0.871
OCTA	SVP inferior vessel density (%)	49.16 [48.37, 49.95]	49.72 [48.62, 50.82]	48.19 [47.25, 49.13]	0.56 [-0.90, 2.01]	0.626	-0.97 [-2.19, 0.25]	0.305	-1.53 [-3.01, -0.05]	0.155
OCTA	SVP temporal vessel density (%)	47.43 [46.94, 47.92]	46.53 [45.89, 47.17]	46.75 [45.44, 48.05]	-0.90 [-1.75, -0.06]	0.155	-0.69 [-2.06, 0.69]	0.493	0.22 [-1.25, 1.68]	0.924
OCTA	SVP nasal vessel density (%)	46.67 [46.23, 47.11]	45.73 [44.98, 46.48]	45.71 [44.65, 46.77]	-0.94 [-1.85, -0.03]	0.155	-0.96 [-2.10, 0.18]	0.296	-0.02 [-1.32, 1.29]	0.980
OCTA	SVP central foveal vessel density, C-FOV (%)	20.10 [19.07, 21.13]	20.04 [18.54, 21.53]	21.18 [19.62, 22.73]	-0.06 [-1.93, 1.81]	0.980	1.08 [-0.72, 2.88]	0.431	1.14 [-1.10, 3.39]	0.493

Values are estimated marginal means or adjusted between-group differences with 95% confidence intervals. Estimated marginal means were evaluated at the overall mean age and pooled sex distribution of the analytical sample. Pairwise contrasts were obtained from generalized estimating equation (GEE) models with participant identifier as the clustering variable, an exchangeable working correlation structure, empirical (sandwich) standard errors, and adjustment for age and sex. q-values are Benjamini-Hochberg false discovery rate (FDR)-adjusted p-values across all pairwise contrasts within each prespecified parameter family. C, controls; C-FOV, central foveal vessel density; F, fellow eyes after unilateral optic neuritis; GCIPL, ganglion cell-inner plexiform layer; mRNFL, macular retinal nerve fiber layer; N, never-ON eyes; OCT, optical coherence tomography; OCTA, optical coherence tomography angiography; pRNFL, peripapillary retinal nerve fiber layer; SVP, superficial vascular plexus.

Supplementary Table S4. Exploratory minimum detectable effect analysis for selected fellow-versus-never-ON contrasts.

Parameter	Adjusted difference Fellow-Never-ON [95% CI]	Adjusted contrast SE	FDR-adjusted q	MDE for 80% power
GCIPL mean (µm)	-3.95 [-7.15, -0.76]	1.63	0.020	4.57
pRNFL total (µm)	-10.79 [-18.64, -2.95]	4.00	0.015	11.21
pRNFL temporal (µm)	-2.42 [-12.15, 7.30]	4.96	0.626	13.90
mRNFL mean (µm)	-3.34 [-6.75, 0.07]	1.74	0.062	4.87
SVP total vessel density (%)	-0.09 [-1.09, 0.90]	0.51	0.963	1.43
SVP superior vessel density (%)	-0.30 [-1.70, 1.10]	0.71	0.871	2.00
SVP inferior vessel density (%)	-1.53 [-3.01, -0.05]	0.76	0.155	2.12
SVP temporal vessel density (%)	0.22 [-1.25, 1.68]	0.75	0.924	2.10
SVP nasal vessel density (%)	-0.02 [-1.32, 1.29]	0.67	0.980	1.86
SVP central foveal vessel density, C-FOV (%)	1.14 [-1.10, 3.39]	1.15	0.493	3.21

Adjusted differences are derived from age- and sex-adjusted GEE models with participant-level clustering. The minimum detectable effect (MDE) was calculated for 80% power at a two-sided alpha level of 0.05 as $(z_{0.975} + z_{0.80}) \times SE$ of the adjusted GEE contrast. MDE values are expressed in the original units of each outcome and are provided to support interpretation of non-significant contrasts. They do not demonstrate equivalence between groups. C-FOV, central foveal vessel density; GCIPL, ganglion cell-inner plexiform layer; mRNFL, macular retinal nerve fiber layer; OCTA, optical coherence tomography angiography; pRNFL, peripapillary retinal nerve fiber layer; SE, standard error; SVP, superficial vascular plexus.
